# Synchrotron phase-contrast microtomography of coprolites generates novel palaeobiological data

**DOI:** 10.1038/s41598-017-02893-9

**Published:** 2017-06-02

**Authors:** Martin Qvarnström, Grzegorz Niedźwiedzki, Paul Tafforeau, Živil Žigaitė, Per E. Ahlberg

**Affiliations:** 1 Department of Organismal Biology, Evolutionary Biology Centre, Uppsala University, Norbyvägen 18A, 752 36 Uppsala, Sweden; 2 European Synchrotron Radiation Facility, 71 avenue des Martyrs, CS40200, 38043 Grenoble, France

## Abstract

Coprolites (fossil faeces) reveal clues to ancient trophic relations, and contain inclusions representing organisms that are rarely preserved elsewhere. However, much information is lost by classical techniques of investigation, which cannot find and image the inclusions in an adequate manner. We demonstrate that propagation phase-contrast synchrotron microtomography (PPC-SRμCT) permits high-quality virtual 3D-reconstruction of coprolite inclusions, exemplified by two coprolites from the Upper Triassic locality Krasiejów, Poland; one of the coprolites contains delicate beetle remains, and the other one a partly articulated fish and fragments of bivalves.

## Introduction

Many coprolites are comparable to small and underexplored Konservat-Lagerstätten (sedimentary deposits with exceptional fossil preservation) in which undigested food remains, including soft tissues, preserve better than in the host rock^1^. The exceptional preservation is linked to a phosphate-rich microenvironment which favours early bacterially-induced phosphatization and lithification^2, 3^; examples of described coprolite inclusions comprise vertebrate soft tissues (e.g. muscle tissue and hairs)^3, 4^, parasites^5^ and many biomineralized tissues^6–8^. Data from such inclusions can illuminate palaeoecological relations in ancient ecosystems as well as the physiology and feeding behavior of extinct organisms^7^.

Coprolite contents have previously almost exclusively been visualized through light microscopy of thin sections and by scanning electron microscopy (SEM)^2–7, 9, 10^. These methods have a number of limitations: they require destructive preparation of samples, provide only 2D (or shallow 3D) imaging, reveal limited information about the spatial relationships of the inclusions, and tend to miss many of the inclusions because of poor sampling of the coprolite’s total volume. Studies based on these methods typically underestimate the contents of the coprolites, fail to recover many delicate fossils, and yield little information about the organization of the inclusions.

A few coprolites (and a fossil regurgitate pellet) have been scanned using either medical computed tomography (CT)^11^ or laboratory μCT^12^. X-ray tomographs allow some 3D visualization of bigger objects and large-scale internal structures (e.g. spirals) of the coprolites^11^. However, the scans have low spatial resolution which makes it impossible to image inclusions on a submillimeter scale, and strong beam hardening is also often encountered when scanning fossils with such kind of machines. Laboratory μCT is able to image small objects at high resolutions, but typically yield unsatisfactory contrasts between mineralized tissues in fossil specimens as their contrast mechanism is based on X-ray absorption only. They can also suffer substantially from beam hardening effects, if well-adapted metallic filters and reconstruction algorithms are not used^13–15^.

Synchrotron microtomography has an excellent track record of imaging the internal structure of fossils with exceptional quality and sensitivity^13, 14^, even on relatively large specimens^16, 17^. Especially, the use of propagation phase contrast effect can reveal structures that are completely invisible using X-ray absorption only^18, 19^. The phase contrast effect derives from detecting phase shifts of the beam emerging through the sample, instead of only recording the decreased beam intensities due to the X-ray absorption of the sample^20^. This results in a much higher sensitivity (about 1000 higher in the energy range used in this study), which becomes extremely useful since mineralized fossils exhibit low absorption contrasts^13^.

This study presents the first application of PPC-SRµCT to coprolites. We describe the contents of two coprolites (ZPAL AbIII/3401 and 3402; Institute of Paleobiology, Polish Academy of Sciences, Poland) from the rich Upper Triassic (upper Carnian) locality of Krasiejów (Poland)^21^. The fossil remains from Krasiejów represent two ecological communities – a so-called lake community (including dipnoan and ganoid fishes, the temnospondyl *Metoposaurus*, the phytosaur *Paleorhinus* and various invertebrates), and a terrestrial community (including small reptiles, the small dinosauriform *Silesaurus*, the large ‘rauisuchid’ *Polonosuchus* and the aetosaur *Stagonolepis*), with the large temnospondyl *Cyclotosaurus* probably inhabiting the lake shore^21^.

**Figure 1:**
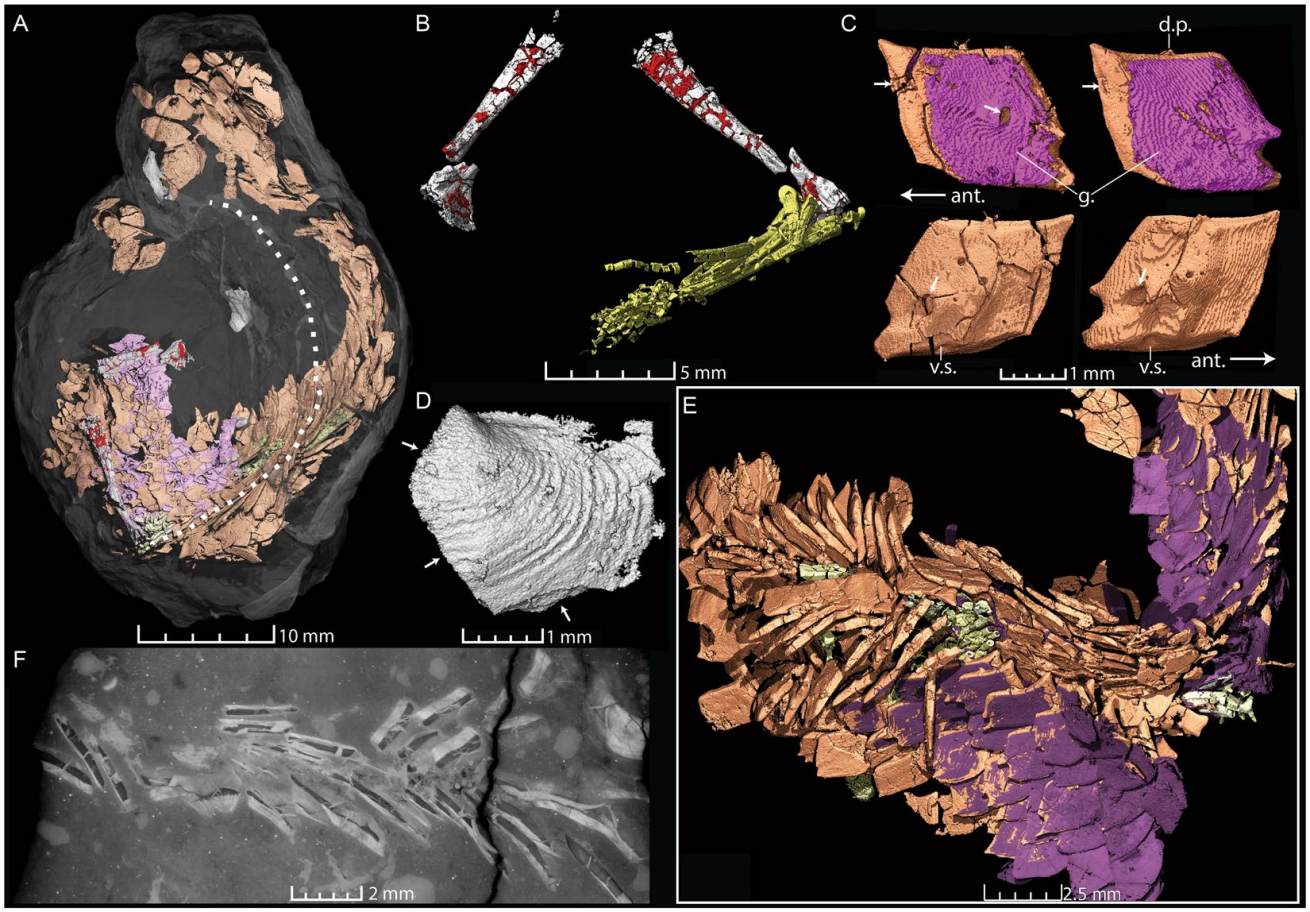
Semi-articulated fish with ganoid scales, probably a redfieldiid, from coprolite ZPAL AbIII/3401. (**A**) The entire coprolite (transparent) with internal fish remains colored as follows: orange – bony part of fish scales; purple – ganoine on the exterior part of the scales (and “infilling” material in cracks); greenish yellow – lepidotrichia of the fins; white – bone and bivalve shells; red – infilled empty spaces in pelvic girdles. The dotted line indicates the inner margin of the spiral coil. (**B**) Ventral view of the left and right pelvic girdles of the fish with the right pelvic fin attached to the girdle. (**C**) Examples of two fish scales of the lateral line in exterior (top) and interior (below) views. The arrows indicate lateral line canal and pore openings. (**D**) Exterior view of a fragmented bivalve shell with arrows indicating surfaces of rupture. (**E**) Close-up of articulated fish scales and fin lepidotrichia. (**F**) Virtual thin section “thick slab” of fish scales and lepidotrichia (visible in the center of the image). Abbreviations: ant. – anterior; d.p. – dorsal peg; v.s. – ventral socket.

Coprolite ZPAL AbIII/3401 (46 mm long, 33 mm wide at maximum width) is incomplete, and appears to have a spiral morphology consisting of one big coil (Fig. 1A). Fish remains and bivalves are visible on its exterior, especially on broken surfaces. 3D-segmentation of PPC-SRµCT data reveals a partly articulated actinopterygian fish (Fig. 1). Fin rays, scales and bones are commonly fractured, folded and sheared from processing in the coprolite producer’s digestive tract, but even delicate structures remain partly articulated. For example, left and right pelvic girdles are preserved in close proximity (Fig. 1B), one with the pelvic fin still attached. Their morphology is very similar to that of the extant actinopterygian *Amia* ^22^.

Abundant ganoid scales constitute the majority of the inclusions. In part, the scales overlap one another in approximately natural articulation (Fig. 1), with for example several aligned lateral line scales, implying that dermal soft tissue survived the digestive tract of the coprolite producer and still held the biomineralized structures together in the faecal mass. Scale preservation ranges from practically intact to very fractured and degraded.

Fish remains in Krasiejów are usually found as isolated scales and teeth, although partly articulated ganoid fishes are also known from the locality. It remains unclear if they belong to a single species, but some have provisionally been attributed to the redfieldiid *“Dictyopyge” socialis* (Berger, 1843)^21^. The ganoid fish from the coprolite is not determinable to genus, because it lacks skull bones, but the squamation is compatible with a redfieldiid.

The coprolite producer was evidently a relatively large aquatic predator. Highly fragmented bivalve shells are also present in the matrix of coprolite A (Fig. 1D), implying that the producer was a durophagous animal that preyed on both fish and mollusks. The spiral shape of the coprolite and the folded fish remains suggest that the gut had a spiral valve, a structure seen in chondrichthyans and some bony fishes^23–25^, but not in tetrapods. No remains of larger chondrichthyans are known from Krasiejów, and the coprolite is very different from the typical tightly coiled spiral coprolites of chondrichthyans. Additionally, injuries matching the dental morphology of lungfish have been recorded on bivalves from another Late Triassic locality in Poland^26^. Together these characteristics and comparisons suggest that the coprolite was most likely produced by the large dipnoan (lungfish) *Ptychoceratodus*, which is known from Krasiejów^27^.

**Figure 2:**
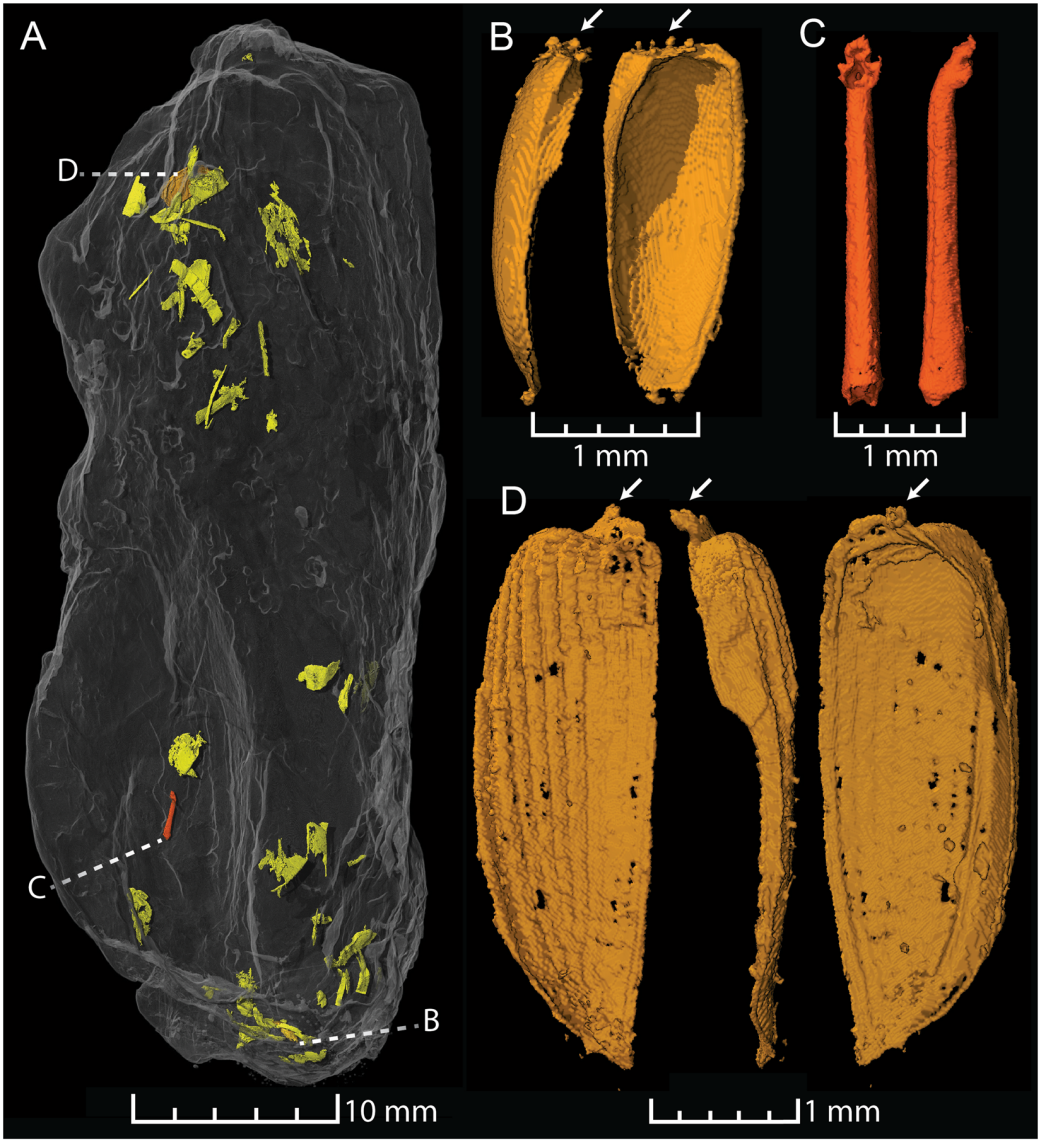
Insect inclusions in coprolite ZPAL AbIII/3402. (**A**) The entire coprolite (semi-transparent) and small fossil insect/arthropod inclusions colored in: brick red – insect tibia; gold – beetle elytra; greenish yellow – other arthropod inclusions. (**B**) Ventral and lateral views of a small beetle elytron. (**C**) Ventral and lateral views of an insect tibia, probably belonging to a beetle. (**D**) Beetle elytron (probably belonging to a beetle of the Polyphaga suborder) in dorsal (left), lateral (middle) and ventral (right) views. The white arrows indicate the articulatory roots of the elytra.

Coprolite ZPAL AbIII/3402 (53 mm long, 23 mm wide) is elongated and non-spiral. Two small and near-complete beetle elytra were found in the matrix. These are preserved in great morphological detail with, for example, the delicate structures of the articulatory roots still intact (Fig. 2). The bigger of the two elytra (3.9 mm long and 1.2 mm wide) displays longitudinal striae (Fig. 2D); a character typically found in beetles of the Polyphaga suborder but that also evolved convergently in the extinct family Ademosynidae. A similar elytron to this specimen was described as *Argentinocupes pulcher* from Argentinian deposits of comparable age^28^. The smaller specimen (2.3 mm long and 0.9 mm wide), has a smooth outer surface. The taxonomic position of this specimen is dubious, since several groups of beetles display similar smooth and wedge-shaped elytra. However, the elytron does most likely not derive from a member of the Archostemata suborder. The Schizophoridae family is the only group within Archostomata with such elytra, and these display a protrusion on the ventral side^29^, which is missing in the described specimen.

An insect tibia with bent tibial head and characteristic denticulate edging on the flexor side was found in the same coprolite as the elytra (Fig. 2C). The tibia probably derives from a beetle, since it has attachment sites for thick chaetae and tibial spurs, lacks spines, and the apices of the tibial head are widened. The beetle elytra and tibia are found in a mishmash of other fragmented insect remains (Fig. 2A).

Insect remains from Krasiejów are only represented by a few isolated beetle elytra found in association with plant material. One of these has been assigned to a cupedid beetle and is the only previously described specimen with detailed morphological preservation^21^. The elytra described herein represent new beetle remains from the locality, and the tibia represents the first insect fossil that is not an elytron.

Coprolite ZPAL AbIII/3402 was produced by an insectivorous animal that evidently targeted small beetles as prey items, but the size of the coprolite suggests that the coprolite producer was a fairly large animal (with a scat diameter comparable to, for example, recent coyotes), and not as small as a modern-day insectivorous lizard or mammal.

The above examples show that PPC-SRμCT is a very powerful method when applied to coprolites. Its advantages can be summarized as follows:

1. *The scans are nondestructive*. Precious or rare coprolites can be studied using PPC-SRμCT as it neither requires destructive preparation nor modifies the coprolite’s chemical composition. It may also be advantageous to virtually reconstruct the inclusions before destructive analyses (e.g. geochemistry) to target the most interesting parts of the coprolite and minimize the loss of information.
2. *It allows high-quality 3D visualization of preserved objects*. Fossil inclusions in coprolites are generally three-dimensionally preserved and often with very little distortion of the original shape (e.g. Fig. 2). This is linked to the early lithification of coprolites and holds often true even in facies which normally display a large degree of flattening of fossils (e.g. shales). As such, there is a gain of information if three-dimensionally preserved fossils are analyzed in all dimensions.
3. *It is possible to visualize the 3D organization of inclusions* (articulated inclusions or the context in which delicate inclusions are found) *and coprolite architecture* (e.g. inner organization of spirals or core-cortex differentiation).
4. *The entire coprolite content can be imaged in high quality*. This allows for the capture of rare and delicate structures (Fig. 2) and for definitive statements about the presence of inclusions, given that these provide an X-ray contrast and are large enough to be recognized, which is relative to the size of the coprolite if the entire specimen is scanned.
  The occurrence of certain inclusions in coprolites potentially allows a statistical evaluation, which is relevant to palaeobiological questions such as diet, parasitism, and the identification of coprolite makers. It is also possible to make quantitative measurements of features such as gas bubbles and inclusions.
5. *The internal structures of the inclusions may be studied*. For example, in the scales of the fish specimen described above, the ganoine layer can be clearly distinguished, as can the canals of the lateral line and vascular supply. PPC-SRμCT has been used to extract life history data from fossil teeth and bones (e.g. from the early tetrapod *Acanthostega*)^30^, and there is no reason why similar information should not be retrievable from coprolite inclusions representing prey animals. This will make it possible to add yet another aspect to the reconstruction of ancient food webs.
6. *Discovery of new fossils*. Since coprolites act as small konservat-Lagersätten (cf. amber), we expect that many rare and new organisms will be discovered with this increased ability to study the inclusions by PPC-SRμCT.
7. *Palaeoecological interactions in ancient ecosystems may be uncovered*. The two coprolites treated herein derive from the same locality and depositional environment, but from two animals with very different ecology and feeding habits (an aquatic piscivore versus a terrestrial insectivore). The application of PPC-SRμCT to a larger number of coprolites from the same locality can potentially enable reconstructions of trophic food webs and other palaecological aspects (e.g. parasitism) of entire ecosystems. This will be the scope of a future study of the rich coprolite assemblage from Krasiejów.

## Methods

The coprolites were scanned using propagation phase-contrast synchrotron microtomography (PPC-SRμCT) at beamline ID19 of the European Synchrotron Radiation Facility (ESRF) in Grenoble, France. The coprolites were scanned in half acquisition mode (i.e. the center at rotation was set at the side of the camera field of view, resulting in a doubling of the reconstructed field of view), in vertical series of 5 mm. The propagation distance, i.e. the distance between the sample on the rotation stage and the camera, was set at 2800 mm. The camera was a sCMOS PCO edge 5.5 detector, mounted on an optical device bringing an isotropic voxel size of 6.54 μm, and coupled to a 1000-μm thick GGG:Eu (Gadolinium gallium garnet doped with europium) scintillator. The beam produced by a W150 wiggler (11 dipoles, 150 mm period) with a gap of 48 mm was filtered with 5.6 mm aluminum and 5 mm copper. The resulting detected spectrum had an average energy of 110.8 keV. Each sub scan was performed using 6000 projections of 0.08s each over 360 degrees.

The reconstructions of the scanned data were based on a phase retrieval approach^31, 32^. Ring artefacts were corrected using an in-house correction tool^33^. Binned version (bin2) were calculated to allow faster processing and screening of the samples since the full resolution data was quite large. The full resolution data can be used to obtain higher detail level for the identified inclusions when necessary. The final volumes consist in stacks of 16 bits TIFF images (in total 3,666 slices for coprolite A and 4,340 slices for coprolite B) that were subsequently imported and segmented in the software VGStudio MAX version 3.0 (Volume Graphics Inc.).

Region of interests (i.e. masks or subdivided parts of the total volume) were mostly created by using the region growing tool. Region growing is a tool for segmentation that selects connected voxels with similar gray scale values. The degree of difficulty in segmenting certain inclusions with this tool is related to the contrast between the inclusion of interest and the coprolite matrix as well as its connection to other inclusions (or areas with secondary mineralization) with similar gray scale values. For example, the fish scales were sometimes difficult to isolate one by one both because they were overlapping, and due to the heterogeneity of the matrix. Some of the beetle remains were also in unnatural conjunction making region growing not always straightforward. All visible inclusions of interest were nevertheless possible to isolate and segment using region growing and multiple small thresholds based on voxels with different gray scale values.
